# Sensor Fusion-Based Vehicle Detection and Tracking Using a Single Camera and Radar at a Traffic Intersection

**DOI:** 10.3390/s23104888

**Published:** 2023-05-19

**Authors:** Shenglin Li, Hwan-Sik Yoon

**Affiliations:** Department of Mechanical Engineering, The University of Alabama, Tuscaloosa, AL 35487, USA; sli90@crimson.ua.edu

**Keywords:** sensor fusion, roadside camera and radar, traffic monitoring, Kalman filter, vehicle detection and tracking, intelligent traffic system

## Abstract

Recent advancements in sensor technologies, in conjunction with signal processing and machine learning, have enabled real-time traffic control systems to adapt to varying traffic conditions. This paper introduces a new sensor fusion approach that combines data from a single camera and radar to achieve cost-effective and efficient vehicle detection and tracking. Initially, vehicles are independently detected and classified using the camera and radar. Then, the constant-velocity model within a Kalman filter is employed to predict vehicle locations, while the Hungarian algorithm is used to associate these predictions with sensor measurements. Finally, vehicle tracking is accomplished by merging kinematic information from predictions and measurements through the Kalman filter. A case study conducted at an intersection demonstrates the effectiveness of the proposed sensor fusion method for traffic detection and tracking, including performance comparisons with individual sensors.

## 1. Introduction

The growing number of vehicles and increasing traffic congestion pose global challenges such as extended wait times, a rise in car accidents, environmental pollution, and higher fuel consumption. To address these issues, considerable effort has been made to develop effective traffic control systems, particularly in large cities and areas prone to traffic congestion [1,2,3]. To make optimal, condition-based decisions, a traffic control system requires accurate traffic information, including real-time vehicle locations and traffic flows. Collecting and analyzing these data are crucial for optimizing traffic management and reducing negative impacts on the environment, safety, and overall quality of life.

Traditionally, inductive-loop detectors have been utilized to provide real-time traffic flow information. However, these sensors can only detect vehicles within a small detection zone, providing limited data. Moreover, traffic may be disrupted during sensor installation under road surfaces. To overcome these drawbacks, advanced sensors such as cameras, radars, and LiDARs are increasingly employed in traffic control applications. These sensors enable the collection of comprehensive, real-time traffic data, including precise vehicle location, type, and trajectory information. Furthermore, different sensors can provide complementary data, which can be combined to overcome individual sensor limitations and produce more accurate information through sensor fusion. Consequently, research on sensor fusion algorithms has gained popularity, as it presents an opportunity to improve the accuracy of traffic state estimation by integrating data from multiple sensors.

The sensor fusion algorithm proposed in this paper consists of three main components: object detection and classification, data association, and object tracking. First, for object detection and classification using a camera, the high accuracy and real-time performance of the well-known algorithm You Only Look Once (YOLO) have made it a popular choice in recent years [4]. Also, a radar module featuring built-in vehicle detection and classification algorithms on the hardware level, produces detected vehicle information without additional data processing. As the camera and radar have separate coordinate systems, it is essential to unify their coordinates and obtain a list of detected vehicles in the same coordinate system. Object classification serves as an auxiliary function to help to improve tracking algorithm decision-making. Second, although traditional data association algorithms, such as Probability Data Association (PDA) [5] and Joint Probability Data Association (JPDA) [6], can achieve good results for tracking performance, the probability calculation requires significant computational resources as the number of detected objects increases. In contrast to Probability Data Association algorithms, the Hungarian algorithm efficiently solves the assignment problem by minimizing the matching distance, without directly comparing all the possible pairs. Thus, the Hungarian algorithm is applied for data association in the proposed method [7]. Lastly, since the results of the camera and radar are presented in the same Cartesian coordinates and the transformation is linear, the tracking algorithm uses a standard Kalman filter to predict the motion of the detected vehicles [8] rather than using a more sophisticated extended Kalman filter (EKF) [9] or unscented Kalman filter (UKF) [10]. A weight matrix is created by calculating the distance between the predictions of the Kalman filter and the measurements of each sensor, and then the Hungarian algorithm is applied to find the best correlation using the weight matrix. With the integration of these three components, more efficient and effective vehicle tracking is expected to be achieved for traffic-monitoring applications using two different types of sensors. It is the first time that the three selected components are systematically integrated to improve vehicle tracking in a traffic network.

This paper is organized as follows: Section 2 reviews the relevant literature and state-of-the-art research. Section 3 details the object detection and classification using the camera and radar sensors, with data alignment offering a solution for unifying the results from different sensors in the same space. Section 4 presents the proposed sensor fusion algorithm framework and explains the vehicle motion estimation, data association, and vehicle tracking processes. Section 5 provides validation results, and Section 6 concludes with a discussion.

## 2. Related Works

Recently, various approaches in multi-sensor fusion for traffic-monitoring applications have been developed. Based on the level of abstraction, sensor fusion algorithms can be categorized into three primary approaches. The first approach is based on the integration of raw data collected using multiple sensors. For example, stereo vision was used to perform object detection based on a disparity map created by combining information from two cameras [11,12]. Other examples include the combination of loop detectors and Bluetooth measurements [13,14]. The second approach considers the features obtained from each sensor individually and then combines these features to perform detection. In [15], a fusion approach that combines different features from a LiDAR and a camera was presented. In these two prevalent sensor fusion approaches, the sensor fusion is based on combining raw data or concatenating features from different sensors. However, it may be challenging to perform sensor fusion in certain situations where one of the sensors does not function normally or the environmental conditions do not allow a particular type of sensor to perform properly [16]. To overcome these difficulties, in the third approach, each sensor performs detection independently, and the detection results of multiple sensors are combined according to their accuracies and uncertainties. In this manner, the detection system can operate with adequate robustness under a variety of operating conditions. In [17,18,19], sensor fusion algorithms that combine detection results obtained independently from radar, LiDAR, and camera are presented. These fusion algorithms produce reliable results and demonstrate sufficient robustness in most situations. However, the prohibitive development and maintenance costs have hindered the widespread adoption of this technology in real-world applications [20].

Several state-of-the-art methods within the third general approach have been proposed for accurate vehicle localization without relying on expensive sensors. One such method combines multiple sensors, including GPS, IMU, and a single camera [21]. This system employs a Kalman-filter-based sensor fusion algorithm to integrate the multi-sensor data and estimate the vehicle’s position and trajectory. However, due to the different data formats of the various sensors, the system requires a more sophisticated extended Kalman filter for data integration. This, in turn, increases the development and computational costs associated with the system, potentially posing a significant drawback for real-time traffic control applications. Another example utilizes camera sensors for vehicle detection and tracking [22]. This method is specifically designed for the real-time estimation and monitoring of highway traffic flow, employing several deep learning techniques and a unique data association algorithm. However, relying solely on vision-based sensors poses challenges when used in inclement weather conditions. Other studies have leveraged deep learning and association algorithms to estimate and monitor traffic flow using camera and radar sensors [23,24]. While employing multimodal sensors can improve the vehicle localization accuracy of these methods, the multiple steps required, including pre-calibration, object matching, and parameter optimization, make them complex and computationally intensive.

In this paper, a new sensor fusion algorithm is proposed based on the third general approach. By taking advantage of the complementary characteristics of different sensor types, such as millimeter-wave and vision-based sensors, the algorithm achieves a higher precision that would be difficult to attain using a single sensor type. This presents a cost-effective solution that can be used to improve traffic safety and efficiency. A novel fusion method, consisting of three components, is introduced, which can systematically incorporate multiple sensors. This renders the algorithm even more versatile than the existing ones that utilize only two sensors. Furthermore, the Hungarian algorithm, in data association, can be further optimized by adding parameters that reduce errors in mismatched data associations. Additionally, a differential GPS is employed as a reference for the quantitative assessment of the performance of the proposed sensor fusion algorithm.

## 3. Object Detection and Classification

Cameras and radar sensors both possess distinct strengths and weaknesses in terms of their detection accuracy, detection range, and robustness to environmental changes. Vision-based sensors, such as cameras, are highly effective in accurately detecting and recognizing objects up to 100 m away. However, beyond this range, the camera accuracy gradually declines, and at distances exceeding 150 m, accurate object recognition becomes nearly unachievable. In contrast, radar sensors can provide longer-distance measurements and are known for their robustness in low-light and adverse weather conditions. While radar sensors might be affected by rare environmental factors such as lightning, their performance is generally more reliable under challenging conditions compared to cameras. To leverage the advantages of both types of sensors, object detection and classification are performed independently by the camera and radar sensors, and the results are combined through a fusion algorithm.

### 3.1. Camera-Based Object Detection and Classification

The camera-based object detection algorithm is based on YOLO due to its high accuracy and real-time performance [25,26]. This research used its fourth version, YOLOv4, which consists of darknet Convolutional Neural Networks as the backbone, Spatial Pyramid Pooling (SPP) [27] and Path Aggregation Network (PANet) [28] as the neck, and YOLOv3 [29] as the head. As explained in the authors’ previous publication, YOLO draws rectangular boxes around vehicles detected in video images [26]. The size of the rectangular boxes depends on the proximity of the vehicles to the camera, as the vehicle size in the image increases when they get closer to the camera. Since only the centers of the vehicles in the image coordinates are converted into 3D world coordinates through coordinate transformation and used as the vehicle locations, the scaling of the vehicle size is not a concern. This entire procedure is described in detail in the referenced work. The camera-based object detection aims to obtain the locations of all the detected vehicles so that this information can be used in the fusion process. In contrast to many sensor fusion techniques, the camera sensor utilized in this research extracts appearance information from images solely for classification, rather than for tracking purposes, for two primary reasons. First, this helps to increase the processing speed of the overall sensor fusion system. Since the goal of this research is to develop a light system that can process the sensor fusion algorithm in real time, an enhanced processing speed is highly desirable. Second, although a tracking algorithm based solely on kinetic information without visual features may cause an ID-switching problem, the radar sensor can significantly mitigate this issue. As a result, the camera sensor provides only a list of detected vehicle locations.

The YOLO algorithm employed in this study for vehicle detection and classification was pretrained with the COCO dataset, which is a larger-scale object detection, segmentation, and captioning dataset for computer vision projects [30]. The camera-based classification model can recognize several vehicle types, including cars (sedans and SUVs), trucks, and motorcycles. The classification information can help to improve data association, and different types of vehicles are utilized to select different fusion parameters in the data association algorithm.

### 3.2. Radar-Based Object Detection and Classification

**Figure 1:**
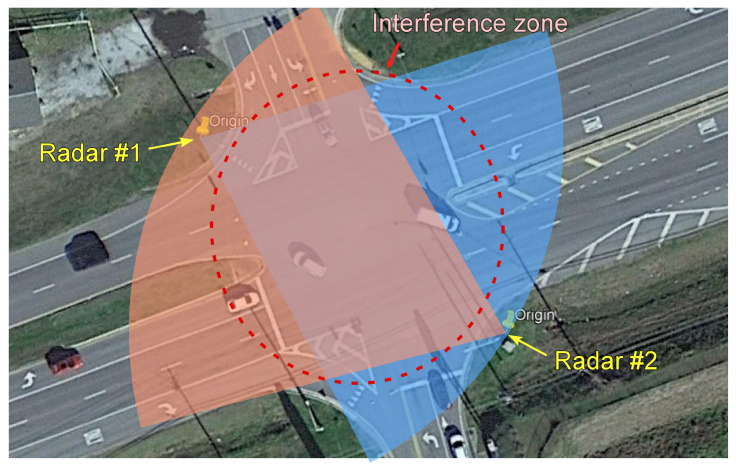
Two radars installed at a traffic intersection.

In this study, a commercially available radar (Model iSYS-5220 from InnoSenT, Donnersdorf, Germany) was used for vehicle detection at a traffic intersection. As shown in Figure 1, two radars are installed to monitor two approaching lanes each situated at an intersection. For optimal object detection, the radar employs proprietary signal processing techniques designed for a long-range and wide-horizontal view. The enhanced coverage ensures the improved detection of moving and stationary objects compared to the camera sensor. However, due to the interference of the two nearby radars, the detection accuracy near the intersection area is not reliable, causing false positives. The radar can classify only two types of vehicles based on the size of the vehicles: cars (sedans and SUVs) and trucks. Similar to the output of the camera-based object detection system, a list of detected vehicle locations is the output of the radar-based detection system.

### 3.3. Data Alignment

**Figure 2:**
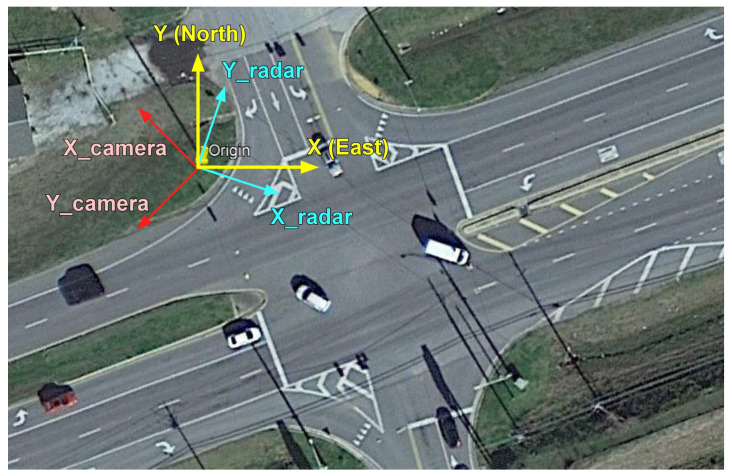
Two sensor coordinate systems and unified coordinate system.

Multiple sensors are usually not placed in the same location, and thus, data alignment is required by matching the coordinate systems of the different sensors. Since both the camera and radar are installed on the same traffic light pole in this research, their coordinate origins are the same. Consequently, one only needs to rotate their coordinates towards the common north–east (Y–X) coordinates for coordinate system matching. After the coordinate system matching, all calculations of the fusion algorithm are performed in the north–east (Y–X) coordinate system. The camera coordinates, radar coordinates, and north–east coordinates are shown in an aerial view map (from Google) in Figure 2.

**Figure 3:**
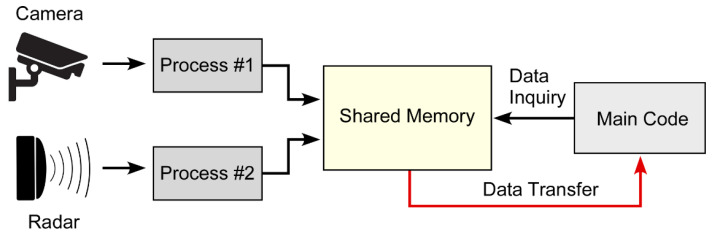
Approach for mitigating network latency using shared memory to store multiple sensor data.

In sensor fusion applications, accessing remote sensors via a network cable can pose challenges due to communication system and network latency. The synchronization of multiple sensor data points can be affected by significant communication delays [31]. Furthermore, sequential accessing of sensor data on the host computer may cause additional non-synchronization errors. A potential solution for addressing these issues is to use the ‘multiprocessing’ module in Python to store the most recent sensor data in a shared memory and then access the shared memory in every iteration. While this approach may not fully resolve all the timing issues, it can leverage the available computing power to mitigate latency problems. The basic concept is shown in Figure 3.

## 4. Sensor Fusion Algorithm

To enhance the vehicle detection capability, a fusion algorithm was developed to optimally combine the vehicle detection results obtained from the two sensors. The proposed sensor fusion algorithm consists of three main steps: vehicle motion estimation, data association, and vehicle tracking.

### 4.1. Vehicle Motion Estimation

A Kalman filter is used in the motion estimation model to track target vehicles detected by the camera and radar sensors. The Kalman filter consists of two phases: prediction and update.

In the prediction phase, the state vector for each target vehicle is calculated as follows:

(1) $$X=\left[x,y,\dot{x},\dot{y}\right]$$

where x and y are the center location of the target vehicle, while $\dot{x}$ and $\dot{y}$ represent the longitudinal and lateral velocity all in the global coordinates, respectively. The estimation for each target vehicle at the current time step, t, using a constant-velocity model is obtained using a kinematic formula as follows:

(2) $${\hat{X}}_{t}=F_{t}{\hat{X}}_{t-1}$$

(3) $${\hat{P}}_{t}=F_{t}{\hat{P}}_{t-1}F_{t}^{T}+Q$$

where ${\hat{X}}_{t}$ represents the mean prediction of the target vehicle location, ${\hat{P}}_{t}$ is the covariance matrix that represents the uncertainty in the estimated state, $F_{t}$ is the state transition matrix, and Q is the system noise matrix. The state vectors of the sensor measurements are given by:

(4) $$Z_{c}=\left[x_{c},y_{c}\right]$$

(5) $$Z_{r}=\left[x_{r},y_{r},\dot{x_{r}},\dot{y_{r}}\right]$$

where $Z_{c}$ and $Z_{r}$ represent the camera and radar measurement vectors, respectively. In the state vectors, $x_{c},y_{c}$ are the vehicle location measured by the camera, and $x_{r},y_{r},\dot{x_{r}},\dot{y_{r}}$ are the vehicle location and velocity measured by the radar.

**Figure 4:**
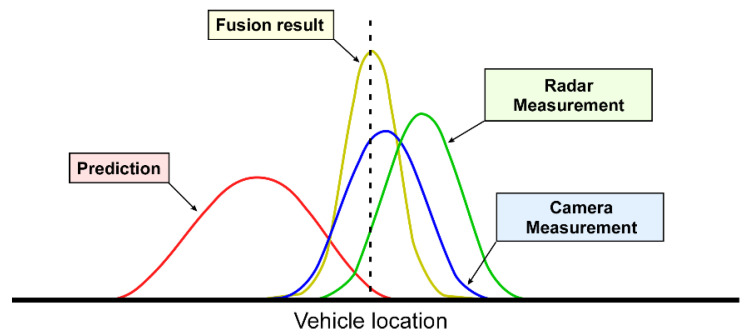
Conceptual process of sensor fusion.

During the update phase, sensor measurements are used to refine the prediction for the target vehicle. Note that the units, scale, and variables of the sensor measurements may not be the same as those for the prediction for the target vehicle. To match these different quantities, the prediction for the target vehicle is projected to the sensor measurement spaces using the following equations:

(6) $$\mu_{i_{t}}=H_{i_{t}}{\hat{X}}_{t}$$

(7) $$\Sigma_{i_{t}}=H_{i_{t}}{\hat{P}}_{t}H_{i_{t}}^{T}$$

where $\mu_{i_{t}}$ and $\Sigma_{i_{t}}$ represent the projected mean and covariance matrices obtained using the estimation in the prediction stage, and $H_{i_{t}}$ is the sensor measurement transition matrix. The index i represents the two different sensors: the camera and radar. The Kalman filter assumes that the predicted state and two measurements exhibit a Gaussian distribution. The next step is to reconcile the difference between the predicted state and the measurements of the two sensors, i.e., the camera and radar. Then, the optimal estimation can be obtained by multiplying the probability density function of the predicted state and the two measurements [32]. Mathematically, the multiplication of these three probability density functions still yields a Gaussian distribution. There are two different ways to multiply the probability density functions of the predicted state and two measurements, depending on the order in which the measurements are multiplied to the predicted state. Both orders were tested, and the results were found to be quite similar. Consequently, only the results from one order are presented in the validation section below. As an example, the derivation of the equations is presented here for the case where the camera measurement is multiplied first and the radar measurement is multiplied next. After multiplying the camera measurement, the state and covariance of the target vehicle, with camera correction, can be written as:

(8) $${\hat{X}}_{t}^{\prime }={\hat{X}}_{t}+K^{\prime }\left(Z_{c_{t}}-H_{c_{t}}{\hat{X}}_{t}\right)$$

(9) $${\hat{P}}_{t}^{\prime }={\hat{P}}_{t}-K^{\prime }H_{c_{t}}{\hat{P}}_{t}$$

(10) $$K^{\prime }=\frac{{\hat{P}}_{t}H_{c_{t}}^{T}}{H_{c_{t}}{\hat{P}}_{t}H_{c_{t}}^{T}+R_{c}}$$

where $K^{\prime }$ is a matrix called the Kalman filter gain that minimizes the covariance, and $R_{c}$ is the measurement noise of the camera. Using the same multiplication method, the state and covariance of the target vehicle with radar correction can be written as:

(11) $${\hat{X}}_{t}^{\prime \prime }={\hat{X}}_{t}^{\prime }+K^{\prime \prime }\left(Z_{r_{t}}-H_{r_{t}}{\hat{X}}_{t}^{\prime }\right)$$

(12) $${\hat{P}}_{t}^{\prime \prime }={\hat{P}}_{t}^{\prime }-K^{\prime \prime }H_{r_{t}}{\hat{P}}_{t}^{\prime }$$

(13) $$K^{\prime \prime }=\frac{{\hat{P}}_{t}^{\prime }H_{r_{t}}^{T}}{H_{r_{t}}{\hat{P}}_{t}^{\prime }H_{r_{t}}^{T}+R_{r}}$$

where $R_{r}$ is the measurement noise of the radar. Once the updated Equations (11) and (12) are calculated, the system transitions into the prediction phase at the next time step, and the algorithm repeats itself. This sensor fusion process is illustrated in Figure 4, where each curve represents the probability distribution of the measured or predicted vehicle locations.

### 4.2. Data Association

Data association involves the assignment of target vehicle predictions to the corresponding sensor measurements. In this research, the data association is performed using the Hungarian algorithm. The Hungarian algorithm provides an efficient way to associate predicted and measured vehicle locations by minimizing the total sum of distances, J, between the predicted and measured vehicle locations, represented in the following equation:

(14) $$J=\sum_{i}\sum_{j}C_{i,j}D_{i,j}$$

where i is the index for the predicted vehicle locations, and j is the index for the measured vehicle locations. Additionally, C is the cost matrix representing the Euclidean distance between various combinations of predicted and measured vehicle locations such that its element, $C_{i,j}$, can be calculated as:

(15) $$C_{i,j}=\sqrt{{\left(x_{p,i}-x_{m,j}\right)}^{2}+{\left(y_{p,i}-y_{m,j}\right)}^{2}}$$

where $x_{p,i}$ and $y_{p,i}$ denote the i-th predicted vehicle location, and $x_{m,j}$ and $y_{m,j}$ denote the j-th vehicle location measured by the sensors. If the i-th row is associated with the j-th column, $D_{i,j}=1$, and otherwise, $D_{i,j}=0$.

Additionally, four conditional statements are applied using the following parameters: the maximum matching threshold, lateral threshold, sensor boundary, and oversized truck identification. First, the maximum matching threshold is employed to reject assignments of measurements to predictions that are too far away. Second, when the lateral distance between the measurement and prediction exceeds the lateral threshold value, indicating that the two vehicles are in different traffic lanes, the assignment is rejected. Third, the sensor boundary is used to apply different weights to the sensors’ trustworthiness depending on the location of the vehicles with respect to the boundary. The radar sensor should be given a higher weighting for a detection beyond 80 m, since the camera cannot recognize objects in this range. Conversely, the camera sensor should be trusted to a greater degree for a detection within 80 m due to interference issues between the two radar sensors near an intersection. Finally, if a sensor detects that the target vehicle is an oversized truck, different matching parameters will be used for this target.

The sensor fusion algorithm effectively manages short-term occlusions by relying solely on the camera. For extended occlusions, the radar assists the camera in tracking the object, as it demonstrates a superior performance in distinguishing an occluded vehicle from the one in front of it. However, in certain cases where a vehicle remains entirely hidden by other vehicles for an extended duration, neither sensor can detect it. In such instances, the sensor fusion algorithm loses the target vehicle’s trajectory and treats it as a new target when it reappears in a subsequent time step.

### 4.3. Vehicle Tracking

Each sensor can detect vehicles within a specific area with high accuracy. By using multiple sensors, the vehicle-tracking performance and localization accuracy can be enhanced by compensating for the deficiencies of individual sensors. Many tracking algorithms use both kinematic states and appearance features [33]. However, there exists another type of method, called the Simple Online and Realtime Tracking algorithm (SORT), which relies solely on kinematic states for object tracking [34]. In this study, a new tracking algorithm based on SORT was developed, using only the kinematic state to fuse the Kalman filter’s predictions with the measurements from the two sensors.

When a vehicle is detected by either sensor, a tracker is created within the tracking algorithm. For the prediction phase of the Kalman filter, the constant-velocity model approximates the tracker locations at the next time step. The Hungarian algorithm is applied to correlate the tracker predictions with the measurements from the first sensor. The tracker locations are then corrected using the measurements from the first sensor following the update phase of the Kalman filter. The results obtained after fusion with the first sensor’s measurements can show one of three cases: matched trackers correlated with the first sensor’s measurements, unmatched trackers not correlated with the first sensor’s measurements, and unmatched detections from the first sensor that are not associated with the existing trackers. The second sensor’s measurements correct these combined results of matched and unmatched trackers using the same Hungarian algorithm.

**Figure 5:**
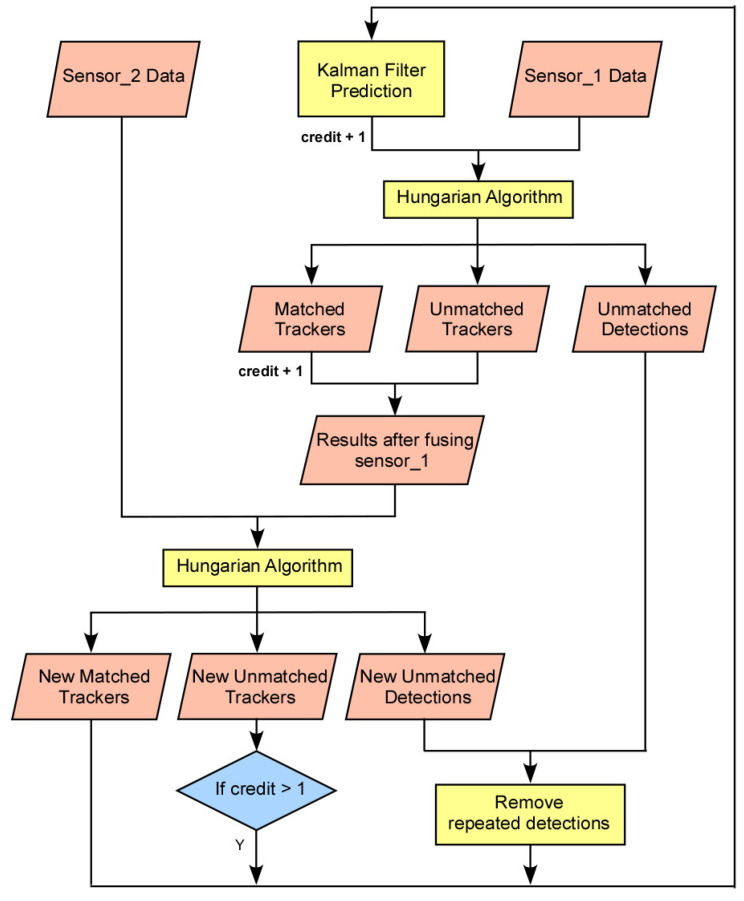
Flowchart for Vehicle Tracking Algorithm.

After fusion with the second sensor, the results still consist of three cases: new matched trackers, new unmatched trackers, and new unmatched detections. In the first case, new matched trackers, which are correlated with either both sensors or the second sensor alone, remain in place. In the second case, new unmatched trackers are not associated with the second sensor. Trackers not associated with either sensor and those that have a credit number no greater than one are filtered out. In the third case, new unmatched detections from the second sensor are not associated with the existing trackers. Finally, the repeatedly unmatched detections from the first and second sensors are removed by a filter, and new, unrepeated detections from both sensors are obtained. These three components constitute the fusion result at the current time step and serve as the input for the next time step. The algorithm is explained in greater detail in the flow chart shown in Figure 5.

In the Hungarian algorithm, the maximum matching threshold rejects assignments if the Euclidean distance between a tracker and a measurement exceeds a predetermined threshold value. When either sensor detects a tracker, one credit is added to its count. If the tracker’s credit count is greater than one, this indicates that the vehicle was detected in the previous time step and was also detected by either of the sensors in the current time step. These trackers are retained to prevent the loss of detections from either sensor.

The constant-velocity model in the Kalman filter is not an ideal predictor of the location of an object, since a real vehicle’s velocity changes continuously. Consequently, the covariance of velocity should be initialized with a large value to minimize reliance on the predicted velocity value. The initial velocity value is empirically set to be slightly lower than the average velocity of the vehicles in the dataset so as to achieve a better performance.

## 5. Experimental Results

The proposed sensor fusion algorithm was assessed using a real dataset collected from two sensors positioned at a traffic intersection. A camera and radar sensor were mounted on a traffic light to monitor a state route with a speed limit of 55 mph. The algorithm’s accuracy was evaluated using three metrics. First, the vehicle localization performance in single-vehicle scenarios was assessed. Second, the vehicle-counting accuracy was measured. Finally, the detection and tracking accuracy of the proposed algorithm was evaluated using multiple object tracking (MOT) metrics [35].

### 5.1. Vehicle Localization Performance

**Figure 6:**
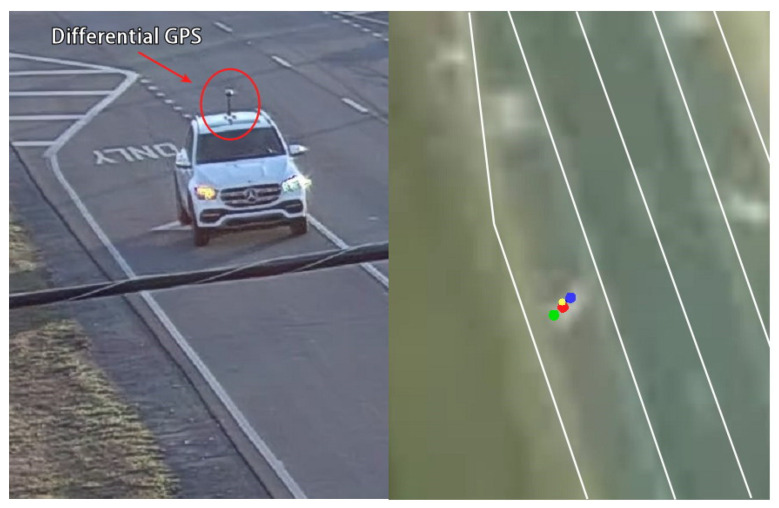
Vehicle equipped with a differential GPS receiver and its location in an aerial view map.

The vehicle localization performance at a traffic intersection was quantitatively evaluated using a differential GPS as the reference. A vehicle equipped with a high-accuracy differential GPS receiver was driven through an intersection at approximately 30 mph, as illustrated in Figure 6. The figure depicts the moving vehicle, along with its location in the world coordinates. The red, blue, and green dots represent the vehicle location measured by the differential GPS, camera, and radar, respectively. The yellow dot represents the sensor fusion result. For the analysis, over one hundred data points were collected in each lane.

**Figure 7:**
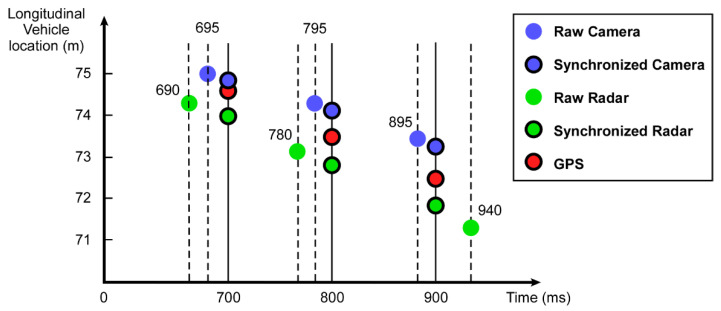
Example of sensor data synchronization for longitudinal vehicle locations using linear interpolation.

The camera records videos at approximately 20 frames per second, with each frame being processed using the YOLO and a vehicle center error correction algorithm to determine the vehicle’s location. The radar and GPS detect vehicles approximately 10 times per second (10 Hz). To synchronize the vehicle detections made asynchronously by the camera, radar, and GPS, the detected vehicle locations are linearly interpolated at a common sampling time, as illustrated in Figure 7. Since the vehicle velocity does not change significantly over short periods of time, the linear interpolation yields highly accurate results with minimal errors.

Table 1 presents the vehicle localization performance of the camera, radar, and the proposed sensor fusion algorithm, respectively. In the table, the lateral location errors correspond to those perpendicular to the vehicle’s travel direction, while the longitudinal location errors correspond to those parallel to the vehicle’s travel direction. Considering the fact that the standard lane width in the United States is 3.66 m, the proposed sensor fusion algorithm’s average lateral error of 0.3 m demonstrates its ability to accurately localize vehicles within their respective traffic lanes. Although the radar’s results after the use of the interpolation method are marginally better than those of the proposed sensor fusion algorithm, the latter remains valuable, as it can address issues such as detection loss during brief periods or difficulties in adverse weather conditions, which the radar sensor may encounter.

Currently, there exist few publications on vehicle detection and tracking using roadside sensors for traffic-monitoring applications. One such method, presented in [21], employs a sensor fusion algorithm that combines data from multiple sensors using an extended Kalman filter algorithm, resulting in a lateral error of 0.53 m, longitudinal error of 1.19 m, and combined Euclidean error of 1.43 m. Another method, proposed in [36], uses multiple cameras to construct 3D bounding boxes around vehicles to determine the vehicles’ locations. The results showed an average localization error of 1.81 m in the Euclidean distance using a differential GPS as a reference and 1.68 m in the Euclidean distance using a drone as a reference. Compared to those previous studies, the sensor fusion algorithm proposed in this research achieves a higher accuracy using only a single camera and radar, with a 0.30 m lateral error, 1.01 m longitudinal error, and 1.06 m Euclidean distance error.

### 5.2. Vehicle Count Performance

The vehicle-counting accuracy for each lane was quantitatively evaluated in a heavy traffic scenario using camera-based, radar-based, and sensor fusion approaches. The camera recorded a two-minute video at 20 frames per second, generating 2400 still images, while the radar captured data at 10 Hz. The proposed sensor fusion system processes high-resolution images with a resolution of 3072 × 1728 pixels at 25 frames per second, utilizing YOLO, center error correction, and sensor fusion algorithms on a 2080 Ti NVIDIA graphics card. To assess the accuracy, the vehicles in each still image were manually counted and used as a ground truth reference. Table 2 presents the vehicle-counting accuracies for camera-only detection with a Kalman filter, radar-only detection with a Kalman filter, and combined camera-and-radar detection using the sensor fusion algorithm.

The table clearly demonstrates that the sensor fusion algorithm outperforms the individual sensors in all cases regarding vehicle detection and counting accuracy. A momentary loss of vehicle detection, caused by internal sensor noise and external environmental effects, negatively impacts the vehicle counting performance of each sensor. Consequently, the average accuracies of camera-only and radar-only methods were 91.36% and 88.47%, respectively. However, by combining the outputs of both sensors, the sensor fusion algorithm could reduce the number of instances of missed vehicle detection, thereby increasing the average vehicle-counting accuracy to 93.32%. Errors occurred only when both sensors missed vehicle detection simultaneously, which was rare.

The vehicle-counting accuracy of all three methods gradually increased from the left lane to the right lane. These results can be attributed to the clearer visibility of the vehicles and the reduced occlusion as the view shifted from the further left lane to the closer right lane. The vehicle-counting accuracy of the sensor fusion algorithm ranged from 87.34% to 97.71%, which is comparable to the accuracy range of 70.58% to 99.63% reported in the literature [22]. However, the sensor fusion algorithm proposed in this study is simpler, offering an advantage over the more complex systems.

### 5.3. Vehicle-Tracking Performance

The collected two-minute-long dataset is divided into two traffic scenarios: light and heavy. Table 3 shows the tracking performance results for the three methods: camera-only detection using a Kalman filter, radar-only detection using a Kalman filter, and the sensor fusion algorithm, all applied to both scenarios. In the table, “#Image” represents the total number of images used in the analysis. “#IDS” indicates the number of instances when an ID switches from one tracked object to another previously tracked object. Meanwhile, “#Vehicle” represents the total number of detected vehicles in each scenario. Using Multiple Object Tracking (MOT) metrics [35], the multi-object tracking accuracy, MOTA, can be calculated as:

(16) $$\mathrm{MOTA}=1-\frac{\Sigma_{t}{FN}_{t}+{FP}_{t}+{IDS}_{t}}{\Sigma_{t}GT}$$

where FN denotes the instances of false negatives in each image, FP represents the instances of false positives in each image, and GT refers to the ground truth number of vehicles in each image. In Table 3, it is shown that the number of ID switches for the sensor fusion algorithm is far less than those of the single-camera and single-radar methods for both traffic scenarios. This can be attributed to the fact that the two different sensors can complement each other, minimizing the number of instances where both sensors miss the vehicle detection. Consequently, the sensor fusion algorithm can provide better precision and a more robust solution in a variety of situations.

## 6. Conclusions

This paper presented a novel sensor fusion algorithm that utilizes a single camera and radar. The algorithm combines vehicle detection data from both sensors, accurately determining the location and trajectory of detected vehicles in a fast and cost-effective manner. By using YOLOv4 and center error correction algorithms, the camera system identifies the locations and types of detected vehicles based on kinematic information, without relying on appearance characteristics. The radar system detects vehicles using its own signal processing hardware. Subsequently, a Kalman filter predicts the vehicle’s trajectory, while the Hungarian algorithm correlates the prediction with the detections from both sensors. The vehicle classification feature further improves tracking by accurately localizing oversized trucks. The sensor fusion algorithm outperforms the camera-only and radar-only methods, achieving an average Euclidean distance error of approximately 1 m, compared to 1.52 m and 0.93 m for the two other respective approaches. Additionally, the sensor fusion algorithm demonstrates a superior vehicle-counting performance, with an overall accuracy of 93.32%, compared to 91.36% and 88.47% for the camera-only and radar-only approaches, respectively. The algorithm also achieves MOTA metrics of 93.73% and 82.51% for light and heavy traffic scenarios. The advantages of the proposed sensor fusion algorithm include its remarkable accuracy, cost-effectiveness, and high processing speed. Although demonstrated with two sensors here, the sensor fusion algorithm could integrate additional sensors if there is sufficient computational power available. Lastly, the computational speed of the proposed fusion algorithm was tested for real-time application in a traffic control system using an NVIDIA AI board, Xavier, achieving a processing speed of seven frames per second. While this processing speed may not be sufficient for real-time applications such as autonomous driving, it is suitable for traffic monitoring and control applications where decision making typically occurs at intervals of 10 s or longer.

In summary, we obtained three key findings with significant implications for the field of traffic monitoring and control, as follows: First, the proposed sensor fusion algorithm outperforms individual sensors, yielding a more robust and accurate solution. Second, the use of kinematic information, without relying on appearance characteristics, is sufficient for the accurate localization and tracking of vehicles. Lastly, a higher accuracy can be achieved with the proposed sensor fusion algorithm, employing a simpler algorithm compared to other state-of-the-art approaches. Overall, the results of this research have significant implications for the field of traffic monitoring and control. More efficient and effective solutions for managing traffic could be developed by continuing to build upon these findings.

## Figures and Tables

**Table 1:** Localization performance in different traffic lanes.

	Error	Camera Results (m)			Radar Results (m)			Sensor Fusion Results (m)			The Number of Data Points
Lane		Lateral Error	Longitudinal Error	Euclidean Error	Lateral Error	Longitudinal Error	Euclidean Error	Lateral Error	Longitudinal Error	Euclidean Error	
Left		0.92	2.38	2.55	0.34	1.37	1.41	0.49	1.71	1.78	125
Middle left		0.62	1.81	1.91	0.24	0.70	0.74	0.35	0.99	1.06	109
Middle right		0.18	0.75	0.77	0.23	0.81	0.84	0.23	0.89	0.92	147
Right		0.22	0.82	0.85	0.19	0.74	0.76	0.11	0.46	0.47	124
Average		0.49	1.44	1.52	0.24	0.91	0.94	0.30	1.01	1.06	126

**Table 2:** Vehicle counting accuracies of different sensing methods at a traffic intersection. ‘C’, ‘R’, and ‘SF’ represent the number of vehicles counted using different combinations of sensors, while ‘G’ denotes the ground truth number of vehicles.

	Camera Only with Kalman Filter			Radar Only with Kalman Filter			Sensor Fusion		
	C	G	Accuracy	R	G	Accuracy	SF	G	Accuracy
Left lane	319	443	72.12%	370	443	83.73%	386	443	87.34%
Middle left lane	2333	2568	90.87%	2221	2568	86.51%	2368	2568	92.25%
Middle right lane	2573	2730	94.26%	2464	2730	90.27%	2588	2730	94.82%
Right lane	339	349	97.42%	333	349	95.62%	341	349	97.71%
Average			91.36%			88.47%			93.32%

**Table 3:** Vehicle tracking results in light and heavy traffic scenarios.

	Light Traffic Scenarios					Heavy Traffic Scenarios				
	MOTA	#Image	#IDS	#Vehicles	#Vehilces (Ground Truth)	MOTA	#Image	#IDS	#Vehicle	#Vehicle (Ground Truth)
Camera	75.1%	1425	672	7259	8065	59.09%	941	1243	9452	12,287
Radar	83.2%	1425	361	7662	8065	72.26%	941	820	10,397	12,287
Sensor Fusion	93.37%	1425	7	7756	8065	82.51%	941	32	10,996	12,287

## Data Availability

Data sharing not applicable.
